# Perception of Portuguese nurses: clinical supervision and quality indicators in nursing care

**DOI:** 10.1590/0034-7167-2022-0656

**Published:** 2023-08-07

**Authors:** Mafalda Sofia Santos Brás Baptista Sérgio, António Luís Rodrigues Faria de Carvalho, Cristina Maria Correia Barroso Pinto

**Affiliations:** I CUF Academic Center. Lisboa. Portugal; II Escola Superior de Enfermagem do Porto. Porto, Portugal; III Centro de Investigações em Tecnologias e Serviços de Saúde & Rede de Investigação em Saúde. Porto, Portugal

**Keywords:** Nurses, Nursing Supervision, Nursing Assistance, Nursing Audit, Evaluation of the Results., Enfermeros, Supervisión de Enfermería, Atención de Enfermería, Auditoría de Enfermería, Evaluación de Resultados., Enfermeiros, Supervisão de Enfermagem, Assistência de Enfermagem, Auditoria de Enfermagem, Avaliação dos Resultados.

## Abstract

**Objectives:**

to describe nurses’ perception of the influence of clinical supervision on improving quality indicators in nursing care.

**Methods:**

exploratory research with a qualitative approach, carried out with 16 nurses using the focus group. Data processing emerged from lexicographical textual analysis, resorting to Descending Hierarchical Classification and similarity analysis.

**Results:**

80.0% retention of 185 text segments with six-class construction. The words were represented by four graphs (supervisor, audit, care, and process); and three subgraphs (implementation, sharing and knowledge).

**Final Considerations:**

in the perception of nurses, supervision influences quality indicators in nursing care.

## RESUMO

**Objetivos:**

descrever a percepção dos enfermeiros acerca da influência da supervisão clínica na melhoria dos indicadores de qualidade na assistência de enfermagem.

**Métodos:**

pesquisa exploratória de abordagem qualitativa, realizada com 16 enfermeiros utilizando o *focus group*. O processamento de dados emergiu da análise textual lexicográfica, recorrendo à Classificação Hierárquica Descendente e análise de similitude.

**Resultados:**

retenção de 80,0% de 185 segmentos de texto com construção de seis classes. As palavras foram representadas por quatro grafos (supervisor, auditoria, cuidado e processo); e três subgrafos (implementação, partilha e conhecimento).

**Considerações Finais:**

na percepção dos enfermeiros, a supervisão influencia os indicadores de qualidade na assistência de enfermagem.

## RESUMEN

**Objetivos:**

describir la percepción de los enfermeros acerca de la influencia de la supervisión clínica en la mejoría de los indicadores de calidad en la atención de enfermería.

**Métodos:**

investigación exploratoria de enfoque calitativo, realizada con 16 enfermeros utilizando el *focus group*. El procesamiento de datos emergió del análisis textual lexicográfico, recurriendo a la Clasificación Jerárquica Descendiente y análisis de similitud.

**Resultados:**

retención de 80,0% de 185 segmentos de texto con construcción de seis clases. Las palabras fueron representadas por cuatro grafos (supervisor, auditoría, cuidado y proceso); y tres subgrafos (implementación, partición y conocimiento).

**Consideraciones Finales:**

en la percepción de los enfermeros, la supervisión influencia los indicadores de calidad en la atención de enfermería.

## INTRODUCTION

The involvement of nursing teams in the evaluation of care practices and the recognition of levels of excellence reflect the professionals’ understanding of the importance of quality and safety and their impact on patients^(1)^. This process, on the one hand, encourages the development of monitoring strategies and the evaluation of daily practice; and, on the other hand, it promotes personal and professional development through reflection on care practices^(2)^.

It is with a vision of organizational leadership in care practice that a private health group in Portugal, made up of several hospitals that care for medical-surgical patients, continuously promotes a differentiating culture of quality and excellence by applying an audit model to the quality of nursing care in its units.

This model was adapted from Haddad’s work on “Nursing Care Quality - The evaluation process in a public university hospital”, applied at the University Hospital of Londrina (HUL) in the scope of a doctoral thesis^(3)^, with the classification of nursing care quality through indicators, as follows: Desired Quality (≤90.0% and <100.0%), Adequate Quality (≤81.0% and <90.0%), Safe Quality (=80.0%), Minimum Quality (≤71.0% and <80.0%) and Inadequate Quality (<70.0%), resulting from sensitive positivity rates.

This process and results management model considered the quality standards of the International Joint Commission (IJC), Quality Management System (QMS), European Standard EN ISO 9001:2015, Health Quality Standards of the General Directorate of Health (DGS) of Portugal and the professional development model of the Order of Portuguese Nurses. All this allows, as a team, to analyse and reflect in an adequate and clear way on the sensitive positivity indices, to implement improvement actions^(4-6)^ in obtaining the desired quality (≤90.0% and <100.0%).

In this perspective of efficiency and full support in the continuous improvement of the quality of care, the concept of clinical nursing supervision emerges as a facilitating strategy in processes of changing attitudes, behaviours, and practices.

Clinical supervision is strategic in the reflection and transformation of thinking and acting in the construction of care practice. When seen as a formal and context-appropriate process, it implies the interaction of nurses in monitoring, planning, evaluating, and implementing improvement actions. On the other hand, it is essential that it takes place in a systematic and interpersonal environment favourable to learning, knowledge sharing and autonomous and responsible decision-making^(7-9)^.

The adoption of clinical supervision also makes it possible to increase human capital, enhance interpersonal, emotional and professional skills with effective gain for the intervening parties, i.e., the supervisor and the supervisee^(10-12)^.

During the supervisory process, according to pre-defined standards, the more experienced professional or supervisor interacts, shares, monitors and effectively guides a less experienced or supervised professional, facilitating the continuous development of skills, the mediation and implementation of actions promoting sense of responsibility and autonomy^(10-11,13)^.

This dynamic should occur according to a formative approach and not a control one, so that reflection, feedback and interpersonal sharing relationships can occur in the team in order to monitor the needs and adapt or adopt the best practices according to the context, with benefits for the professional, staff and organization^(13-16)^.

However, to legitimize the practice of clinical nursing supervision in organizations, it is necessary to know the organizational culture as well as the personal and professional characteristics of supervisors and supervisees in order to stimulate and deepen reflective practice based on evidence^(11,17)^.

Thus, the clinical supervision processes in nursing demonstrate influence in obtaining desirable results with an impact on nursing care practice, increasing patient satisfaction and safety with the health organization^(18-19)^.

Given the above and taking into account the quality indicator of the audits of nursing care practices that the private health institution performs monthly, which revealed sensitive or non-compliant quality indices with a direct impact on patients, there is a need to reflect with the teams about the institutional model of audits and obtain their perception about clinical nursing supervision as a personal and professional development strategy by monitoring care practices to improve results.

This led us to the study question: Does the implementation of clinical nursing supervision in the teams influence the indicators of nursing care quality for medical-surgical patients in the context of a private health unit?

## OBJECTIVES

To describe nurses’ perception of the influence of clinical nursing supervision as a strategy to improve nursing care quality indicators.

## METHODS

### Ethical aspects

This research was approved by the Ethics Committee of the organization involved. All participants were informed and clarified about the research content. The Informed Consent Form was duly completed and signed by each of the participants and returned to the researchers.

The characterization form of the participants was also completed and returned in a sealed envelope and placed in a proper place. In the study, the confidentiality of the data and the anonymity of the participants as well as the services involved were guaranteed. In this sense, each service was represented by a letter (A and B); and the participants, by the letter N (Nurse).

### Study type

This is an exploratory study with a qualitative approach.

Its development was guided by the Consolidated Criteria for Reporting Qualitative Research (COREQ) guide, recommended for research reports that collect data through interviews or groups. It is an integrated study in a doctoral research project that aims to evaluate the impact of clinical supervision in nursing in raising quality indicators in nursing care for medical-surgical patients in the context of hospitalization in a private health unit.

### Study scenario

The research presented was carried out between August and October 2019. The first stage took place in a private hospital in the region of Lisbon, Portugal, in the medical-surgical hospitalization services, where the model of audits of the quality of nursing care is applied.

This model consists of applying a grid for observation and analysis of records of medical-surgical patients with a hospitalization rate of more than 24 hours, aiming to control and monitor the quality of care provided by the nursing teams.

This model results in evidence of compliance or deviations in procedures that impact care practice, called “positivity indices” and subsequently categorized into quality-sensitive indicators, as follows: Desired Quality (DQ), Adequate Quality (AQ), Safe Quality (SQ), Minimum Quality (MQ) and Inadequate Quality (IAQ). In this way, continuous improvement actions are defined.

### Data sources

The research participants are the nurses who are part of the teams of the medical-surgical hospitalization services. Sample elements were selected for the research in a stratified manner according to the equity of the representation of the services and the performance of the focus group sessions. Inclusion criteria were: being a nurse at one of the two surgical and medical hospitalization services, regardless of professional category (junior, nurse I, nurse II, senior, expert, manager), having at least three years of professional practice in the service and in organization and at least one year of practice in the profession.

### Data collection and organization

For data collection, the focus group methodology was used, building a guide to reflect on the themes: perception of the quality of nursing care, perception of the importance of quality auditing in nursing care, perception of clinical supervision of nursing and perception of the supervisory process and its intervenients. Data collection was carried out by a trained researcher, who was familiar with the hospital environment where the investigation took place. The focus group was held in two moments in order to meet the requirements of this methodology^(20)^, which recommends a maximum of six to eight participants per moment.

The focus group moments took place in a comfortable and spontaneity environment for the participants, at a previously defined place and time. Moderation was carried out by one of the researchers, knowledgeable about the profession and the organizational culture, and lasted an average of about 90 minutes. Each participant was guaranteed the opportunity to intervene in the focus group. To record the speeches, audio and image recordings were used for further data analysis^(20-21)^.

### Methodological procedures

The contents of the focus group moments were transcribed and the software IraMuTeQ - *Interface de R pour les Analyzes Multidimensionnelles de Textes et de Questionnaires* (IRAMUTEQR)^(22-23)^ was used as a tool to support data processing in the qualitative study.

IraMuTeQ is developed in the Python language with functionalities provided by the statistical software R, which allows statistics on texts obtained from interviews, documents, among other sources, and transforms them into lexicographic textual analyses according to the Descending Hierarchical Classification (DHC) and similarity.

The software is a data processing tool, not a research method, which makes the results tools for exploring and associating the material under study.

### Data analysis

To carry out the lexical analysis, the textual corpus was prepared, on which the software proceeded to cuts every 40 characters, forming the analysed text segments. To reflect the homogeneity of the corpus, we considered the analysed text segments with retention ≧75%. As a criterion for including words in the classes, all words with a frequency greater than twice the average occurrence in the textual corpus were considered.

According to the DHC of the groupings, the text segments were successively partitioned according to the co-occurrence of lexical forms, originating lexical classes with percentage values represented in a dendrogram.

Association with class was considered by the chi-square value (χ^2^)≧3.84 with a cut-off point (f) greater than or equal to twice the mean frequency (f>12) and p<0.0001, which represent strong correlations between words; for the similarity analysis, the results of the relationships and connectivity of objects in a given set were interpreted^(24-25)^.

Statistical analysis was divided into two phases. The first consisted of a preliminary exploratory approach focusing on thematic structural analysis to measure the relationship between the content’s “audit” and “clinical supervision”. The second phase corresponded to the monothematic structural analysis with the purpose of deepening the understanding of the meaning of the phenomena of the study itself.

## RESULTS

The total number of participants from the three services was 16 nurses, of which 12 were female, with an average age of 40 years. Regarding the professional category, they were nurse managers (n=3), nurse I (n=3), nurse II (n=3), junior nurse (n=3) and senior nurse (n=4) and had an average time in service of 16.5 years.

As for the academic degree, nine of the professionals are only licensed, two are licensed and have a postgraduate degree in clinical supervision, and five have a master’s degree and a postgraduate degree in clinical supervision.

**Figure 1:**
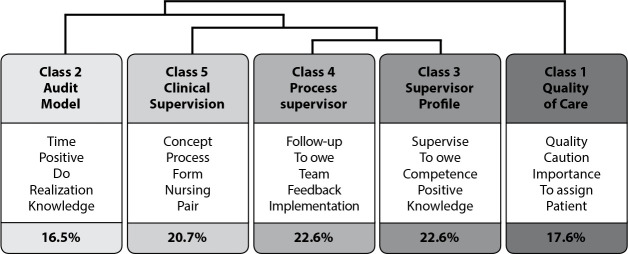
Dendrogram of thematic analysis of classes and their correlations according to Descending Hierarchical Classification, Lisbon, Portugal, 2021

From the analysis of the thematic approach textual corpus, the DHC allowed to obtain 6,979 occurrences presented in 1,088 different ways. Of the 194 text segments, there was a retention of 84.5% with the construction of five semantic classes on the concept of “Clinical supervision” and “Nursing care quality audit model”, represented in the dendrogram (Figure 1).

In the dendrogram, the textual corpus was divided into three subcorpora, in which Class 1 articulates with Class 2; and then these subcorpora associate with Class 5, which relates to Classes 3 and 4, which interact with each other. That is, the *Supervisory Process* and the *Supervisor Profile* are associated with *Clinical Supervision* and integrated into the Nursing Care Quality *Audit Model*.

By applying the chi-square test (χ^2^), in Class 1- *Quality of Care* (17.6%), the words with the highest number of correlations presented are: *quality* (40.0; p<0.0001), *care* (32.1; p<0.0001) and *patient* (34.4; p<0.0001). In Class 2- *Audit Model* (16.5%), the words are *positive* (178.1; p<0.0001) and *accomplishment* (128.9; p<0.0001); for Class 5- *Clinical Supervision* (20.7%), they are *concept* (67.8; p<0.0001) and *nursing* (62.1; p<0.0001).

Finally, in Class 3- *Supervisor Profile* (22.6%), the words are *competence* (7.1; p<0.0001), *positive* (14.6; p<0.0001); and in Class 4- *Supervisory Process* (22.6%), *follow-up* (27.5; p<0.0001), *feedback* (8.7; p<0.0001) and *implementation* (55.1; p<0.0001).

As for the quality of nursing care by class (Table 1), Class 1- *Quality of Care* is perceived as nursing care in accordance with the standards predetermined by the organization, ensuring the safety of patients inserted in the context. For Class 2 - *Audit Model*, in practice this is seen as an important tool in identifying sensitive positivity indices, as it allows implementing actions to improve nursing care.

**Table 1:** Nurses’ perception of the quality of nursing care, according to each class, Lisbon, Portugal, 2021

Classes	Example of participant responses
**Class 1** Quality of Care	*It is the provision of quality care within procedures and standards defined in accordance with current norms and guidelines and in accordance with the patient’s expectations.* (FG1, N1) *It is providing care safely according to scientific knowledge and the needs of the patient and family.* (FG2, N14)
**Class 2** Model of audit	*A tool that identifies what needs to be improved or what needs to be worked on more diligently, to ensure that everyone works the best they know how and that the path to follow is marked out.* (FG2, N10) *Instrument that helps us to improve and that tells us where we are.* (FG1, N7) *These are moments of learning and reinforcement.* (FG2, N16)
**Class 3** Supervisor Profile	*Communicator who likes to teach, relate, lead the other to critical thinking, develop reflection to promote learning. It is knowing how to share and lead to the discovery of knowledge.* (FG1, N4) *Monitoring and guidance of procedures and people, with constructive feedback.* (FG1, N1)
**Class 4** Supervisory Process	*Process developed by nurses for nurses with evidence of learning curve or feedback according to needs.* (FG2, N15) *Developed in a logic of developing skills and in a logic of developing knowledge.* (FG2, N16)
**Class 5** Clinical Supervision	*Lasting learning over time* [...] *anchored in knowledge* [...] *is a logic of sharing professional and personal growth.* (FG1, N8) *Knowledge sharing between supervisor and supervisee in a logic of reflection on sharing values related to the profession and the organization.* (FG1, N7)

Concerning the Class 3- *Supervisor Profile*, nurses identify it as the ability to integrate, monitor and guide new professionals in an assertive, adequate, and competent manner. In Class 4- *Supervisory Process*, this is seen as a strategy for monitoring the daily practice that promotes moments of individual and team sharing. Finally, Class 5- *Clinical Supervision* is identified as an engine for transmitting knowledge, learning, sharing and reflection, which allows the development of skills integrated into practice.

Regarding the similarity analysis, this led to the composition of four central cores represented by the words *care, audit, process, and supervisor*, which denote the perception attributed by nurses to clinical supervision and the organizational audit model.

The branching between the nuclei demonstrates terms that are strongly interconnected, revealing that nurses understand clinical supervision as a facilitating element in the process of *monitoring, feedback* and *reflection* on the quality of nursing care.

In the *Audit* core, the branches with greater connectivity are the *model* and *auditor*, followed by the terms *feedback, guarantee*, and *evaluate*, which are interconnected with the *Care* core with greater connectivity to *quality*, as mentioned in Class 1- *Quality of Care* and Class 2- *Audit Model*.

In the *Process* core, the branches with the greatest connection are clinical supervision with homogeneous branches in terms of *monitoring* and *reflecting*, as mentioned in Class 4- *Supervisory Process* and Class 5- *Clinical Supervision*, in which it is possible to *monitor, reflect* and give *feedback* to the *team* of nursing when implementing the *supervisory process*. Finally, there is the *Supervisor* grouping with greater connectivity in the *profile, competence, follow*-*up, nurse*, and *team branches*, in which three subgraphs emerge to the outside of the axis with the terms: *implementation and obstacle, sharing, personal growth, knowledge, nursing, convey and concept*. This represents the nurses’ perception of the fundamental characteristics of the *Supervisor Profile*, as mentioned in Class 3.

Having ensured the reliability of these results of clinical nursing supervision in improving nursing care, the understanding of the meaning of the phenomenon itself follows.

**Figure 2:**
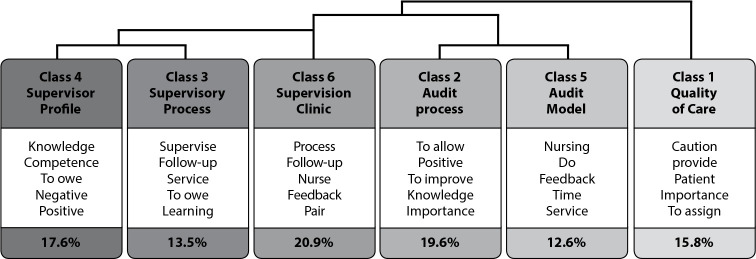
Dendrogram, monothematic analysis of classes and their correlations according to Descending Hierarchical Classification, Lisbon, Portugal, 2021

For the monothematic approach, 6,808 occurrences were analysed, with 1,088 different forms and with a cut-off point f>12. Of the 185 text segments analysed, there was a retention of 80.0%, with the construction of six classes (Figure 2).

In the dendrogram, the textual corpus was divided into four subcorpora, in which Class 1 appears, articulated with Class 2 and 5, related to each other; and Class 6, which articulates with Classes 3 and 4, which are related. That is, the *Supervisory Process* and *Supervisor Profile* are associated with *Clinical Supervision* and are integrated with the *Audit Mode*l and *Process* in nursing care in use in the organization.

Regarding the perception of nurses regarding the quality of nursing care, according to each class (Table 2) and through the application of the chi-square test (χ^2^), in Class 1- *Quality of Care* (15.8%), the words with the highest number of correlations presented are: provide (45.7; p<0.0001), *care* (41.9; p<0.0001) and *patient* (65.3; p<0.0001). For Class 2- *Audit Process* (19.6%), the words are: *allow* (27.5; p<0.0001), *positive* (24.0; p<0.0001) and *improve* (15.9; p<0.0001). In Class 5- *Audit Model* (12.6%): *service* (9.7; p<0.0001) and *nursing* (59.7; p<0.0001).

**Table 2:** Nurses’ perception of the quality of nursing care according to each class, Lisbon, Portugal, 2021

Classes	Example of participant responses
**Class 1** Quality of Care	*It is the provision of care that complies with all the rules and procedures of the institution, based on recent scientific content and updating of professionals.* (FG1, N4) *It is to meet the needs and expectations of patients and the contents that define good practice and quality, providing safe and effective care.* (FG1, N8)
**Class 2** Audit process	*Objectively measuring acts to guarantee and ensure the basic posture of the entire team across the board* [...] *providing moments of learning and better knowledge of processes and services.* (FG1, N8) *It allows giving feedback and having margin of progression, opportunity to improve.* (FG2, N13)
**Class 3** Supervisory process	*They are an engine for transmitting knowledge; reflection facilitates the implementation of improved actions and can be a guiding principle in improving care.* (FG2, N15) *Structured for peers where the supervisor supports and monitors during the provision of care, identifies actions to improve care; also, the supervisee must stipulate meeting times with the supervisor to receive feedback and involvement in the process and results.* (FG1, N6)
**Class 4** Profile of Supervisor	*Be a leader in conflict management with the perspective of professional helping and personal growth by promoting self-growth.* (FG1, N7)
**Class 5** Model of audit	*Having the ability to support, accompany in moments and know how to explain what is happening, stipulate feedback and involvement in the process and result.* (FG1, N6)
**Class 6** Supervision Clinic	*Monitoring within the area of expertise according to the objectives, for professional construction throughout the career.* (FG2, N10) *Global process that allows adapting the learning curve individualized and sustained over time of good practice.* (FG1, N3) *Logic of reflection on the phenomenon in which there is sharing of values related to the profession and organization.* (FG1, N7)

Finally, in Class 4- *Supervisor Profile* (17.6%), the words are *competence* (6.9; p<0.0001), *positive* (15.0; p<0.0001); and, in Class 3- *Supervisory Process* (13.5%), they are *follow*-*up* (11.4; p<0.0001) and *supervise* (13.0; p<0.0001).

In Class 1- *Quality of Care*, related to the concept of quality of nursing care, nurses define it as providing care with clinical safety in accordance with evidence-based practice, with constant updating of professionals’ skills. For Class 2- *Audit Process and Class* 5- *Audit Model*, nurses report that the model is regularly performed by nurses (peers) and is oriented towards a desired quality culture, while the process is oriented towards results of care practices.

In Class 3- *Supervisory Process*, this is translated as a generator of personal and professional learning and a promoter of reflection and sharing on practice in building a path on a par. As for Class 4- *Supervisor Profile*, the nurses mention that this should be recognized by the organization and have a formal and informal view of the process, be motivated, and motivate, support and share learning in a perspective of mutual professional construction.

Finally, in Class 6- *Clinical Supervision*, the nurses consider that it is the transfer of knowledge within their area of expertise, from the supervisor to the supervisee, according to the objectives of the service and the organization and that it is related to monitoring, follow-up, and learning, enabling adequate feedback.

**Figure 3:**
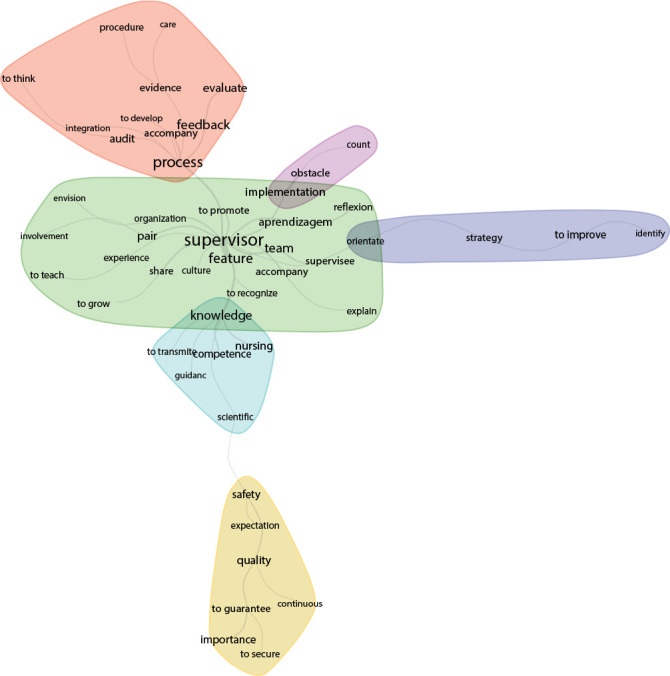
Similarity analysis of the concepts of Clinical Supervision and the Audit Model, Lisbon, Portugal, 2021

As for the similarity analysis, this led to the composition of three central cores represented by the words *Process, Knowledge, and Supervisor*, which denote the nurses’ perception of the clinical supervision strategy in improving the positivity rates of the quality of nursing care resulting from the audits (Figure 3).

The branching between the nuclei demonstrates strongly interconnected terms, which lead to the idea that the clinical supervisor is a professional recognized by the team, possessing specific characteristics, knowledge and skills that allow him, through constructive feedback, to share and monitor their peers in care practice procedures.

In the *Process* core, the branches with greater connectivity are *auditing, monitoring, feedback and evaluating,* which are interconnected with the *Supervisor* core. There is also a homogeneous connection with the terms: *integration, evidence, and care, reinforcing the Supervisory Process in the integration, development and monitoring of peers.*

In the *Knowledge* core, the branches with the greatest connection are competence and nursing; and with a homogeneous connection, the terms *transmit,* and *orientation appear.*

The *Supervisor* core presents greater connectivity in the characteristic, *team, peer and learning branches* and homogeneously for the terms *recognize, promote, share*, and *monitor*. Thus, two subgraphs emerge with the terms: *implementation* and *obstacle*, as well as *guide, strategy* and *improve*, leading to the conclusion that the *supervisor* must adopt strategies and implement supervisory processes in order to overcome obstacles and improve care practices.

## DISCUSSION

The results of the nurses’ perception give visibility to the importance of implementing nursing clinical supervision processes in improving the audit positivity rates, by contributing to the increase in the indicators of the quality of nursing care.

The nurses in the study, with regard to the quality of nursing care, consider that this is a constant in compliance with pre-defined norms and standards based on knowledge and technical-scientific skills in line with the expectations and needs of the patient and family, such as observed in Class 1 - Quality of care and corroborated in the literature^(1,7,12)^.

Regarding the model and audit process in use, the nurses consider them transversal and contextualized tools of care practice that, through positivity indices, identify weaknesses. This makes it possible to develop contextualized improvement actions and sustain the adopted quality indicators, as observed in Class 2 *Audit Process and* Class 5 *- Audit Model*.

At the same time, they attribute importance to the continued normalization of nursing care practice with a direct impact on the quality and safety of patients, which is in line with what is described in the literature^(3,12)^.

It is reported in the literature that supervision, when carried out as an interactive process of monitoring an activity according to a framework, allows ensuring the quality of actions centred on the intervention, as well as the sharing of knowledge between the different stakeholders^(7,15,17-18,26-27)^. This was informed by the nurses in the study, who mention clinical supervision and the supervisory process as a duty and a competency developed by peers, based on sharing knowledge aimed at improving the processes of care practice.

They also add that the supervisory process allows interaction through feedback with an impact on intrapersonal and professional construction and guides towards co-responsibility, understanding and sharing of experiences as observed in Class 6- *Clinical Supervision*, which adds effectiveness to supervision and promotes development of skills and knowledge, also corroborated in the literature^(26-28)^.

In relation to the characteristics of the clinical supervisor, the nurses consider him as a professional recognized by peers and by the organization, holder of concrete and systematized knowledge in the field of clinical supervision and the discipline of the profession, demonstrated in Class 4- *Supervisor Profile*. They also highlight as positive characteristics: assertiveness, communication and leadership as the basis of the relationship of trust, respect, sharing and consolidation of interpersonal relationships; as well as guidance and planning of improvement actions as mentioned in the literature^(8,11-12,14-15,18)^.

Finally, they identify that the supervisor should encourage reflection among peers for emotional management and self-awareness, self-criticism, and self-assessment skills, so that change and cooperation in the supervisory process occurs based on the development of skills that promote new learning with organizational gains. In this sense, the literature states that the supervisor must have theoretical, pedagogical and clinical skills in accordance with the domain of theoretical-cognitive knowledge, experiential and relational know-how that lead to reflection on action in accordance with an organizational culture^(12,17)^.

### Study limitations

Considering that the research was carried out in only one private health unit with hospitalization of medical-surgical patients in Portugal, the application of the supervision model should not be generalized; and yes, contextualized and adapted to the needs of the organization and patients.

However, the methodology can be replicated in other hospital units and services with characteristics similar to those of the participants, allowing the crossing of data and the discussion of the phenomenon.

### Contributions to the field of nursing

It is hoped that the results presented here will support further research aimed at continuing the study on the relevance of clinical nursing supervision as a strategy for gaining personal and professional skills, with a direct impact on the patient and on improving quality indicators of care practice. of nursing.

## FINAL CONSIDERATIONS

The study made it possible to: deepen the perception of nurses regarding the concept of auditing and the concept of clinical nursing supervision; and measuring the impact on quality indicators in nursing care.

We can say that audits are an essential tool in the implementation of continuous improvement processes with a direct influence on patients. In addition, when developing a contextualized model of clinical nursing supervision by peers, it is possible to reflect based on evidence on the practice, guide the monitoring of indicators, implement improvement actions according to the results, establish proximity feedback and induce responsible decision-making, fundamental in professional and organizational development.

This model seems to be the guiding principle for behaviour change, as supervision directly and significantly influences skills at a personal and professional level, being a process that goes beyond nursing theories and concepts.

It is concluded that the research carried out supports the perception of researchers about the application of clinical supervision in nursing, as a strategy for improving quality indicators in nursing care resulting from positivity rates. There is an awareness that change enables nurses to build their professional and interpersonal identity over time, strengthening reflective practice with consequences for nursing care in teams, in the organization and in Nursing as a discipline.
